# Temporary Balloon Occlusion and Dialysis Strategy in Arteriovenous Graft-Induced High-Output Heart Failure

**DOI:** 10.1016/j.jaccas.2026.110204

**Published:** 2026-09-30

**Authors:** Muhammad Junaid Mustafa, Sehar Rana

**Affiliations:** a Punjab Institute of Cardiology, Lahore, Pakistan; b Azra Naheed Medical College, Lahore, Pakistan

The case report by Bhardwaj et al^1^ regarding the successful diagnosis and management of high-output heart failure (HOHF) and secondary pulmonary hypertension (PH) caused by a long-standing arteriovenous graft provides valuable insights that we read with great interest. The authors present a clear and accurate illustration of a challenging clinical scenario requiring both diagnostic precision and interdisciplinary teamwork.

The case describes a patient whose HOHF and PH resolved after ligating a 22-year-old arteriovenous graft, following diagnostic temporary balloon occlusion during right heart catheterization. What makes this case instructive is the strategy used in resolving the primary issue. Arteriovenous access is considered a lifeline for patients with end-stage renal disease, making clinicians hesitant to permanently sacrifice a graft. The decision to perform acute balloon occlusion during right heart catheterization provided immediate evidence of causality. The drop in mean pulmonary artery pressure and pulmonary capillary wedge pressure during temporary occlusion unmasked the true hemodynamic burden imposed by the high-flow circuit. This objective, real-time assessment offers multidisciplinary teams the justification needed before committing to an irreversible surgical intervention.

Moreover, the successful transition to peritoneal dialysis marks a critical strategy for managing end-stage renal disease in patients with severe cardiac strain. By transitioning to peritoneal dialysis, the team successfully removed the strain without compromising renal replacement therapy. This move not only restored the patient's functional mobility—allowing her to transition from walker dependence back to independent ambulation—but also enabled her to be successfully relisted for kidney transplantation.

As Bhardwaj et al^1^ note, HOHF and PH remain underrecognized complications of long-standing vascular access. Although larger prospective efforts, such as the UK COBALT (Cardiorespiratory Optimisation By Arteriovenous fistula Ligation after renal Transplantation) trial, will help clarify objective criteria for access ligation,^2^ this case serves as a timely reminder that high-output cardiac strain is potentially reversible. It provides valuable insight into how cross-specialty collaboration can fundamentally redefine a patient's clinical trajectory.
